# Effects of Pictorial Cues on Reaching Depend on the Distinctiveness of Target Objects

**DOI:** 10.1371/journal.pone.0054230

**Published:** 2013-01-30

**Authors:** Andrea Christensen, Svenja Borchers, Marc Himmelbach

**Affiliations:** 1 Division of Neuropsychology, Hertie-Institute for Clinical Brain Research and Centre for Integrative Neuroscience, Eberhard Karls University, Tübingen, Germany; 2 Section Computational Sensomotorics, Hertie-Institute for Clinical Brain Research and Centre for Integrative Neuroscience, Eberhard Karls University, Tübingen, Germany; Radboud University Nijmegen, The Netherlands

## Abstract

There is an ongoing debate under what conditions learned object sizes influence visuomotor control under preserved stereovision. Using meaningful objects (matchboxes of locally well-known brands in the UK) a previous study has nicely shown that the recognition of these objects influences action programming by means of reach amplitude and grasp pre-shaping even under binocular vision. Using the same paradigm, we demonstrated that short-term learning of colour-size associations was not sufficient to induce any visuomotor effects under binocular viewing conditions. Now we used the same matchboxes, for which the familiarity effect was shown in the UK, with German participants who have never seen these objects before. We addressed the question whether simply a high degree of distinctness, or whether instead actual prior familiarity of these objects, are required to affect motor computations. We found that under monocular and binocular viewing conditions the learned size and location influenced the amplitude of the reaching component significantly. In contrast, the maximum grip aperture remained unaffected for binocular vision. We conclude that visual distinctness is sufficient to form reliable associations in short-term learning to influence reaching even for preserved stereovision. Grasp pre-shaping instead seems to be less susceptible to such perceptual effects.

## Introduction

Grasping objects in our everyday life usually not only involves motor programming of our hand movements towards the target but also the recognition of these objects in order to plan an appropriate action. It had been suggested that visuomotor guidance is predominantly processed in the dorsal (occipito-parietal) stream, while object recognition depends critically on the ventral (occipito-temporal) stream [1]. How these streams of information are interacting to produce a meaningful action is not clear yet and it is still a matter of debate to which extent perceptual judgements affect the programming of grasping movements (for a review see [2]).

The familiar size of an object is a specific pictorial depth cue and depends by definition on object recognition. This learned pictorial information particularly becomes important for action programming when other cues, like binocular depth cues to estimate target distance, are either not reliable or reduced [3]. Only if an object can be identified and its stored representation with its typical size can be accessed then the object’s absolute distance can be computed without stereovision [4]. This concept was discussed as the size-distance invariance hypothesis stating that the perceived size and distance of an object linearly correlate [5]. When grasping under monocular vision, Marotta and Goodale [6] have shown that learned familiar size can be used as a distance cue. Under binocular vision, however, the learned size-distance relationship was not taken into account for action programming in their experiment. They hypothesised that other cues provided by binocular vision were much more reliable to diminish the effect of familiar size. But when binocular depth cues are reduced with monocular vision, the weighting of the remaining familiar size cue might be increased [7]. The visual form agnosia patient D.F., suffering from bilateral lesions in the ventral stream, showed impaired determination of distance under monocular vision while she performed normally under binocular vision (e.g. [8], [9], [10]). Presumably, under monocular conditions pictorial size provides the crucial information for depth perception. Therefore, Marotta et al. [9] concluded that the ability to use these pictorial cues for visuomotor programming relies on an intact ventral stream.

McIntosh & Lashley [11] for the first time have used ‘real’ objects that might have been known by the participants from everyday life and additionally provide a variety of visual cues (e.g., colour, surface pattern) to investigate their effect on motor programming. If familiarity can provide a reliable cue for motor programming even when other binocular cues are available then the effect should be particularly evident if subjects know the objects from interacting with them not only during a few baseline trials but also possibly from pre-experimental everyday experience. Specifically, two types of commonly known matchboxes with different sizes were presented. Participants grasped these boxes during 42 baseline trials at five different distances where each of the matchboxes was mostly presented at one of the five distances. On the last two trials, scaled replicas of the original matchboxes were presented at a distance where the scaled replica projected a retinal image consistent with the original box at a different distance in the baseline trials. McIntosh & Lashley [11] found that subjects over-reached for the smaller replica of the originally larger box at the near distance and under-reached for the large replica of the originally smaller box at the far distance. The effect was amplified in the monocular condition but was also evident and highly reliable in the binocular condition. Based on this surprisingly clear result McIntosh & Lashley [11] concluded, contrary to Marotta & Goodale [6], that the visuomotor system accesses learned knowledge not only when binocular vision is denied, but uses its access to stored object knowledge, presumably mediated by the ventral system, also with full stereoscopic vision.

In order to assess whether the effect observed by McIntosh & Lashley [11] could be simply due to short term memory representations that were formed by associative learning during their experiment or whether the results might have been driven by a ‘real’ familiarity effect, i.e. by stable object representations that were formed by prior experience with the respective objects in everyday life, we repeated the same experiment with geometrical cuboids [12]. These cuboids with exactly the same dimensions as the matchboxes were coloured and unfamiliar to the participants prior to the experiment. For our purposes we replicated the setup and experimental design used by McIntosh & Lashley [11]. In contrast to the original study, we did not find any effects of the size-colour association under binocular conditions although the effect under monocular viewing was almost identical to the one observed in the report of McIntosh & Lashley [11]. Thus, we concluded that indeed the reported familiarity effect was probably not simply due to a short term learning process. However, the stimuli used by Borchers et al. [12] differed in visual complexity from those used by McIntosh & Lashley [11]. The original matchboxes were not only distinguishable by their primary colour but additionally were clearly different in their surface patterns and local contrasts. These additional cues could have influenced their distinctness and thus the reliability weighting determining the impact of the pictorial cues on visuomotor programming. Hence, we now presented the same matchboxes that were used by McIntosh & Lashley [11] to German students that have never seen them before. We expected an effect of familiar size during monocular viewing based on the preceding reports [6], [11], [12]. If we find that the familiar size cue is used under binocular vision as well, then this would suggest that visual complexity and the distinctness of objects increases their reliability weighting for motor computations. However, if subjects still did not use the pictorial cues for programming their grasping movements when binocular vision is available, it would rather suggest that objects need to be highly familiar, and subjects must have prior experience with them in order to affect visuomotor computations when very reliable binocular cues are available.

## Methods

### Participants

Thirty-four German participants (15 females) were tested. Stereoscopic vision was assessed by the screening plates of the TNO stereotest and shown to be normal in 32 subjects. These participants all passed at least the first plate of the TNO and the median depth level that subjects could discriminate was 60 degrees in both visual groups. Two subjects who did not pass the TNO test were excluded from further analysis. All participants had normal or corrected-to-normal visual acuity and were right-handed according to the Edinburgh Handedness Inventory (EDH: [13]). Participants were assigned to two different groups, either to the binocular (B) or to the monocular (M) viewing condition. As assessed by the Porta test, all 16 subjects that were assigned to the monocular condition were right-eye dominant; participants in the binocular condition had either left, right or mixed ocular dominance (Table 1). Mann-Whitney U test found no reliable differences between the groups in age (p = 0.093), stereovision (p = 0.361), or laterality quotient (p = 0.820). All participants have indicated never having seen the matchboxes before. The experiment was approved by the local ethics committee of the University of Tuebingen (reference number: 590/2010 BO2) and conducted in accordance with the 1964 Declaration of Helsinki. All participants gave their informed written consent prior to testing.

**Table 1:** Group assignments and subject information.

Group	N	Sex	Age (years)	Handedness(EHI score)	Eye dominance
B	16	8 f, 8 m	24.9 (4.9)	87.5 (14.4)	3 r, 8 l, 5 b
M	16	7 f, 9 m	27.0 (4.7)	86.9 (15.8)	16 r

Mean values and SD of age and Handedness score. EHI: Edinburgh Handedness Inventory; Eye dominance r: right, l: left, m: mixed ocular dominance.

### Procedure

**Figure 1:**
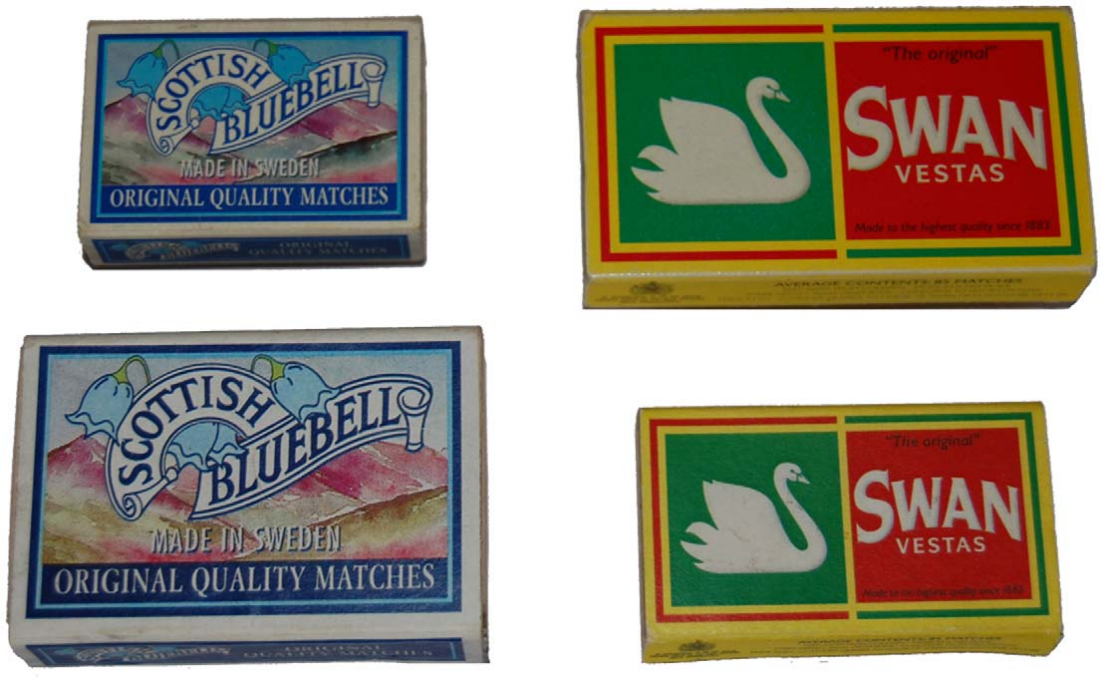
Objects to be grasped. The matchboxes adapted from McIntosh & Lashley [11] that were presented to German subjects. Original matchboxes (upper row) that were presented during baseline trials and their replica used during the last two perturbation trials (lower row). The replica of the Bluebell box was scaled with a factor of 1.25, while the replica of the Swan Vestas box was scaled with a factor of 0.8 compared to the original matchboxes.

We used the same setup that was used by McIntosh & Lashley [11] and Borchers et al. [12]. Subjects sat at a table with a white backdrop at 70 cm distance from the eyes (100 cm x 180 cm) that filled the entire field of view. To avoid distinguishable shadows of the objects in the field of view the room was diffusely illuminated. A chin and forehead rest was fixed to the table to prevent participant’s head movement during the experiment. Each trial started when the LCD shutter glasses (PLATO, Translucent Technologies), that were used to control visual presentation times, turned from opaque to clear. One of two different matchboxes (Fig. 1) was presented at one of five distances (270, 315, 360, 405, 450 mm) directly in front of the eye(s) at eye level. The objects were fixed magnetically to a metal rod, which was not visible to the subject. Earplugs and earphones prevented subjects to hear any potential background noises giving a cue about the upcoming trial, e.g. click sounds while attaching the respective matchbox to the rod. The subjects held a start-point attached to the table between the right index finger and thumb at the beginning of each trial. A tone presented 500 milliseconds after viewing onset cued the participant to reach out and grasp the object, top-to-bottom, between index finger and thumb. After movement onset vision was allowed for 2 seconds. Prior to testing, each subject performed about 8 practice trials in order to familiarise himself/herself with the procedure and the setup. The practice item’s dimensions (110×17×12 mm) were different from the actual test objects and it was presented at random distances.

We used the same matchboxes that were used by McIntosh & Lashley [11] and are common for the Scottish but unknown for the German population (Fig. 1). For the first 42 experimental trials, a small *Scottish Bluebell* (53×36×14 mm) and a big *Swan Vestas* (79×45×13 mm) matchbox was presented in a completely randomised order. For the baseline trials the smaller *Bluebell* box was presented nine times at 360 mm distance (‘near’ baseline trials) and the bigger *Swan* box was presented nine times at 450 mm (‘far’ baseline trials). These baseline trials were interspersed with three repetitions of filler trials for each box at each of the four other distances. On trial 43 and 44 perturbations of the standard matchboxes were applied in counterbalanced order between subjects. Instead of the bigger *Swan* box, a small *Swan* box was presented at 360 mm distance, projecting the retinal image as the original *Swan* box being presented at 450 mm distance but having the same box-height for grasping like the *Bluebell* box during the ‘near’ baseline trials. Vice versa, instead of the original *Bluebell* box, a big replica was presented at 450 mm distance, projecting a retinal image consistent with the standard *Bluebell* box at 360 mm but having the same box-height as the original *Swan* box (Fig. 1).

### Kinematic Data Acquisition and Analysis

**Figure 2:**
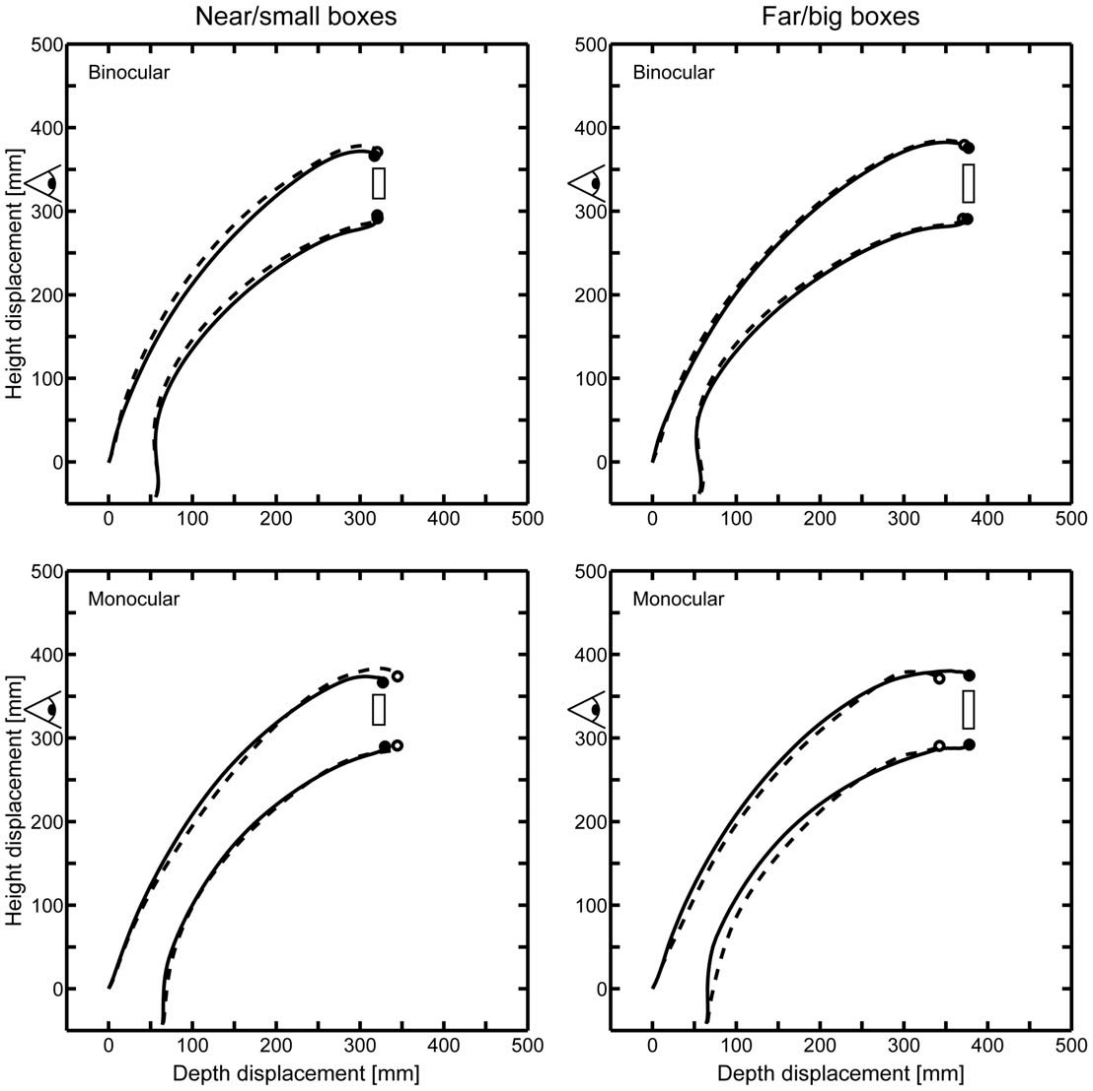
Average trajectories of thumb and index finger. Solid lines and filled circles indicate mean movements and endpoints for the baseline conditions, dashed lines and open circles show the trajectories for the perturbation trials. Upper and lower panels show the mean trajectories of subjects under binocular and monocular viewing condition, respectively. Left and right panels depict the grasping movements to physically near/small and far/big boxes, respectively.

Five infrared light-reflecting markers were attached to the right hand of the subject, at each side of the wrist, half way of the os metacarpale secundum, and to the distal phalanxes of the thumb and index finger. The 3D positions of the movements were recorded with a sampling rate of 120 Hz (Vicon Motion Systems, Oxford, UK). Data was analysed offline using custom software based on Matlab 7.5 (Mathworks Inc., Sherborn, MA, USA). Raw data was smoothed with an averaging window of 10 data points. Movement onset was defined from the tangential speed of the wrist marker using a threshold of 50 mm/s. Movement offset was determined from the acceleration profile of the wrist marker, using the second zero crossing as the endpoint of the trajectory. As in Borchers et al. [12] we have chosen a criterion different from McIntosh & Lashley [11] because most of our subjects produced a pretty smooth transition between grasping the object and taking it from the rod. Therefore, resultant tangential velocity frequently did not fall below 50 mm/s although the object was already successfully grasped. In some trials, the fingers closed already to grope for the cuboid before the end of the transport movement as detected by the acceleration criterion. Such an attempt clearly indicates the participant’s expectation to find the respective object in this place. Therefore, movement end was defined by the acceleration criterion only if, after the MGA was achieved, the grip aperture during the hand transport was never smaller than the final grip aperture at the object itself. Otherwise, movement end was defined by the first local minimum of grip aperture that was smaller than final grip aperture. Only for illustrative purposes, mean trajectories across each group were calculated based on individual movement trajectories that were interpolated yielding 100 data points (Figure 2).

We calculated the depth displacement of the index finger in the x-y-plane at movement offset (DD) and maximum grip aperture (MGA) between index finger and thumb marker. For both parameters we compared the mean of the last three baseline trials for each original matchbox with the respective perturbation trial using the replica of the same physical but with a different associated familiar size as done in Borchers et al. [12].

## Results

During the experiment the participants learned the sizes and the most probable locations of the individual boxes and thus got fooled by the replica in the unexpected, unfamiliar sizes presented at those locations that were used with the highest frequency in the baseline phase. As a result the participants over-reached for the near/small *Swan* boxes and under-reached for the far/big replica of the *Bluebell* box. This effect was observable for both visual groups, although metrically smaller for the binocular group (mean displacement bias: 4.22 mm vs. 26.38 mm). Average trajectories of the thumb and the index finger compared to the perturbation trials are depicted in Figure 2. Please note that here we cannot unequivocally dissociate learned object-size and object-location associations. To some extent both associations can be formed simultaneously during the experiment because the perturbation test location for each object was presented more often than other locations in the baseline phase of the experiment. For the sake of easier readability, we will nevertheless refer to this unknown combination of object-size and object-location associations as ‘familiar size’ instead of ‘familiar size and location’ in the following text.

These qualitative observations were confirmed by our quantitative analysis of the depth displacement (x-y-plane) for the index finger. An overview of the results of our descriptive and statistical analyses is given in Table 2 and 3. We conducted a mixed-model ANOVA with the between-subject factor view (binocular, monocular) and the within-subject factors physical size (near/small, far/big) and familiar size (*Bluebell* box is near/small, *Swan* box is far/big). As expected and in line with our previous experiment [12] both main effects for familiar and for physical size were highly significant. In contrast to the first experiment with the blue and red cuboids there was no overall between-subject effect for the viewing condition [12]. Despite the lack of a main effect of view, the interaction of familiar size and view was significant reflecting an amplification of the familiar size effect in the monocular compared to the binocular viewing condition. To investigate the group differences in more detail we conducted repeated measures analyses for both groups individually (see Table 3). These analyses revealed strong influences of familiar size on the depth displacement for both the binocular and the monocular group, with amplified effect sizes for the monocular viewing condition (partial η^2^ 0.794 vs. 0.479).

**Table 2:** Descriptive statistics of depth displacement (DD) and maximum grip aperture (MGA).

			Binocular				Monocular			
Physical size			near/small	near/small	far/big	far/big	near/small	near/small	far/big	far/big
Familiar			near/small	far/big	far/big	near/small	near/small	far/big	far/big	near/small
DD	Mean		317.45	321.43	377.39	373.27	327.26	343.76	378.01	341.76
	Std		13.95	14.49	13.72	15.57	8.40	14.71	12.56	20.15
	95% CI	Lower	310.02	313.71	370.08	364.97	322.79	335.92	371.32	331.02
		Upper	324.88	329.14	384.70	381.56	331.74	351.60	384.70	352.49
MGA	Mean		96.48	97.50	103.99	104.28	103.79	108.15	108.28	106.58
	Std		6.06	5.90	5.47	5.73	9.26	9.59	10.41	9.58
	95% CI	Lower	93.25	94.36	101.08	101.22	98.86	103.04	102.73	101.48
		Upper	99.71	100.64	106.91	107.33	108.73	113.26	113.82	111.69

Values are presented for baseline and corresponding perturbation trials in mm: baseline near (original Bluebell), perturbation near (small Swan replica), baseline far (original Swan), perturbation far (big Bluebell replica).

**Table 3:** Inferential statistics of depth displacement and maximum grip aperture.

	Depth Displacement (xy)			Maximum Grip Aperture		
within subjects	overall	binocular	monocular	overall	binocular	monocular
	***	**	***	*		*
familiar size	F = 70.13	F = 13.79	F = 57.92	F = 5.11	**n.s.**	F = 5.948
	p<0.001	p = 0.002	p<0.001	p = 0.031		p = 0.028
	***	***	***	***	***	
physical size	F = 448.44	F = 1288.3	F = 49.744	F = 45.67	F = 75.734	**n.s.**
	p<0.001	p<0.001	p<0.001	p<0.001	p<0.001	
	**		*			
familiar size x physical size	F = 7.87	**n.s.**	F = 7.98	**n.s.**	**n.s.**	**n.s.**
	p = 0.009		p = 0.013			
**between subjects**	**Depth Displacement (xy)**			**Maximum Grip Aperture**		
				*		
view	**n.s.**			F = 5.65		
				p = 0.024		
**interaction effects**	**Depth Displacement (xy)**			**Maximum Grip Aperture**		
	***			**°**		
familiar size x view	F = 37.75			F = 3.13		
	p<0.001			p = 0.087		
	***			***		
physical size x view	F = 69.15			F = 19.93		
	p<0.001			p<0.001		
	*					
familiar size x physical size x view	F = 7.63			**n.s.**		
	p = 0.01					

F- and p-values are reported for within and between-subject effects in mixed model ANOVAs including both visual conditions (first columns) and for the individual visual conditions separately (columns labelled binocular and monocular). Values are reported for each effect and variable that yielded at least a statistical trend (p<0.1). Asterisks indicate statistical significance (*p<0.05, **p<0.01, ***p<0.001).

**Figure 3:**
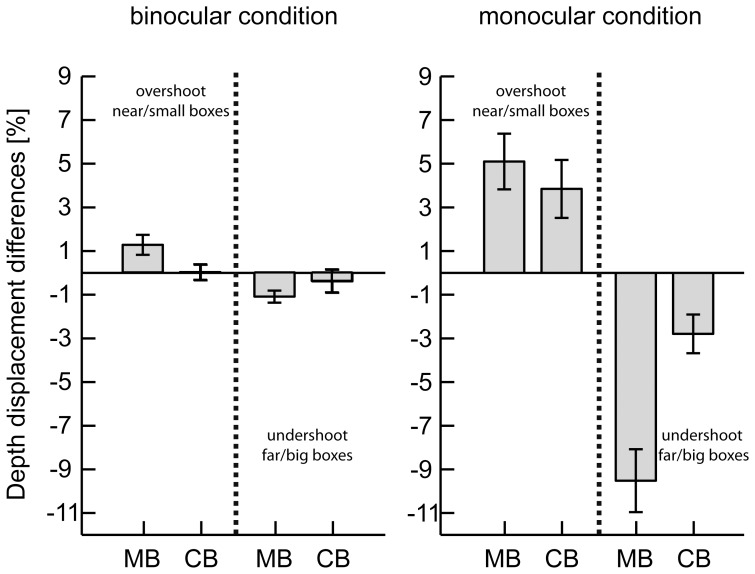
Comparison of the depth displacement bias between experiments. Bars show relative bias in the perturbation trials with respect to the corresponding baseline trials for the experiment with the coloured red and blue boxes (CB) and the matchboxes (MB) in percent. Errorbars indicate standard errors (SE).

To quantitatively test the differences between our new findings, using the matchboxes, and those from the previous study, using coloured cuboids, we calculated the mean shift bias for each condition in both experiments for the perturbation trials with respect to the corresponding baseline trials. The mean biases are depicted in Figure 3. We observed that for all conditions, the monocular and the binocular viewing condition, and for both directions of perturbations the effects were larger for the matchboxes than for the coloured cuboids: participants over-shoot and under-shoot, respectively, for the near/small replica of the *Swan* box and the far/big replica of the *Bluebell* box more than for the corresponding scale replicas of the coloured cuboids.

A 2 (experiment, between-subject)×2 (viewing condition, between-subject)×2 (direction of perturbation, within-subject) ANOVA revealed a highly significant difference between experiments (F(2,61) = 9.09, p<0.001). Further, we observed a significant effect of the viewing condition (F(2,61) = 24.03, p<0.001) in the sense that the overall biases were larger for the monocular condition, and an interaction of viewing condition and experiment (F(2,61) = 5.69, p = 0.005).

To investigate the differences in more detail we calculated two separate ANOVAs for the two viewing conditions. There were significant differences especially for the over-shoot in the binocular (F(1,31) = 4.67, p = 0.039) and for the under-shoot in the monocular condition (F(1,31) = 16.29, p<0.001), with larger effect sizes and partial η^2^ in the matchbox than in the coloured cuboid study (Table 4).

**Table 4:** Outcome of one-factorial ANOVAs of familiar size of reach amplitude.

Distances		F	p-value	Partial η^2^	Effect size	Sample size	Corr. among rep measures	Critical F	Power
Matchboxes	B	13.79	0.002	0.48	0.96	16	0.95	4.54	1.00
	M	57.92	<0.0001	0.79	1.96	16	0.17	4.54	1.00
Coloured cuboids	B	0.48	0.500	0.03	0.18	17	0.87	4.49	0.76
	M	14.09	0.002	0.47	0.94	17	0.48	4.49	1.00

ANOVAs were calculated for both visual conditions and corresponding results of post-hoc power analyses of reach amplitude (distance) were computed with G*power 3. The effect size was computed using the actual partial η^2^ of our data for the binocular and monocular condition. Power analyses are based on the a priorily chosen α error probability threshold of 0.05.

Thus, inspecting the results for the depth displacement leads to the conclusion that the association of size and box was stronger for the matchboxes than for the coloured cuboids.

**Figure 4:**
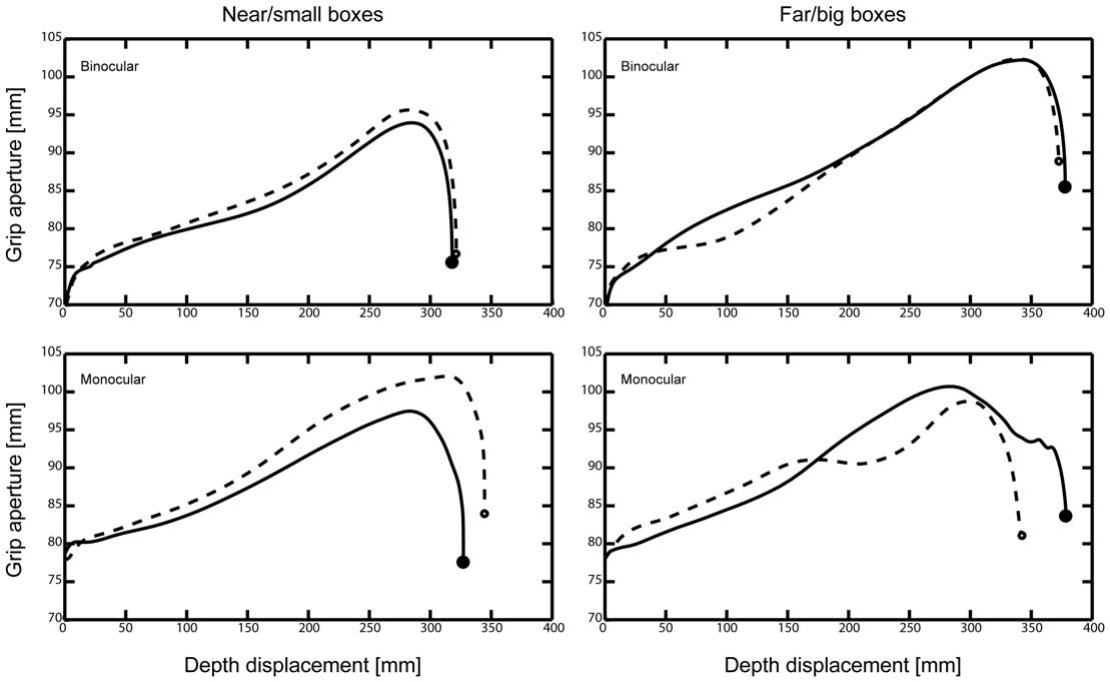
Average evolvement of the grip aperture with increasing depth displacement. Solid lines and filled circles indicate mean movements and endpoints for the baseline conditions, dashed lines and open circles show the trajectories for the perturbation trials. Upper and lower panels show the mean grip apertures of subjects under binocular and monocular viewing condition, respectively. Left and right panels depict the grasping movements to physically near/small and far/big boxes, respectively.

In contrast, the results for the grasping component of the movement showed a different picture. The evolvement of the MGA comparing the baseline with the corresponding perturbation trials is illustrated in Figure 4. The MGA is overall larger for the monocular viewing condition compared to the binocular condition. Participants in the monocular condition were more influenced by the learned object-size association than participants of the binocular group. Especially for the near/small perturbation participants over-scaled their grasp under monocular view.

These observations were confirmed by our statistical analysis of the MGA. Although we observed a significant main effect of the familiar size on the MGA, there was a strong effect of view and a trend for an interaction effect of view × familiar size (Table 2 and 3, Figure 4). To analyse the differences for the viewing conditions in more detail we conducted individual analysis for each subject group. Whilst the familiar size effect was present for the monocular viewing condition under preserved stereovision, familiar size did not influence binocular grasping.

**Figure 5:**
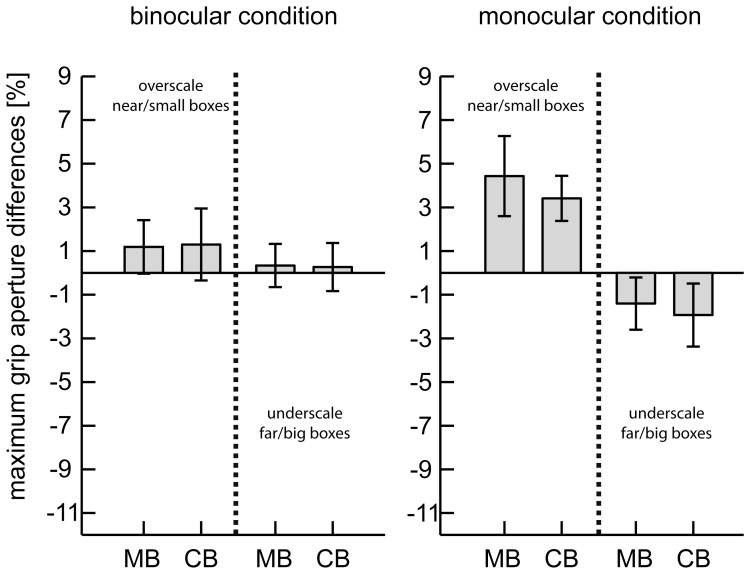
Comparison of the MGA bias between experiments. Bars show relative bias in the perturbation trials with respect to the corresponding baseline trials for the experiment with the coloured red and blue boxes (CB) and the matchboxes (MB) in percent. Errorbars indicate standard errors (SE). Statistical analysis revealed no differences between experiments, neither for binocular nor for monocular condition.

As expected we observed highly significant effects of the physical size on the MGA (η^2^ = 0.604, see Table 3). For the physical size the effects were more pronounced in the binocular condition (7.51 mm differences between large and small boxes; η^2^ = 0.835) compared to the monocular condition (4.49 mm; η^2^ = 0.130) resulting from a general tendency of participants in the monocular condition to overscale the MGA (see also main effect of view in Table 3). To exclude that our null findings for familiar size on grasping in the binocular viewing condition could be attributed to low power of our analysis, we performed post-hoc power analysis using G*power 3 [14]. The results of the power analysis are summarised in Table 5. Based on the effect size of familiar size on the MGA that resulted in a significant outcome in the study of McIntosh & Lashley [11], the post-hoc analysis yielded a power (1-β) of 100% for our study. Based on the effect sizes observed in our sample, the estimated power was 99% for the analysis for the binocular viewing condition (Tables 5). Although the effects are metrically larger for the present experiment compared to the previous study using the coloured cuboids for monocular vision (see Figure 5), statistical testing revealed no difference between experiments neither for the monocular nor for the binocular viewing condition (F(1,31) = 0.4, p = 0.53).

**Table 5:** Outcome of one-factorial ANOVAs of familiar size of MGA.

MGA		F	p-value	Partial η^2^	Effect size	Sample size	Corr. among rep measures	Critical F	Power
Matchboxes Germany	All	5.107	0.031	0.145	0.4118	32	0.85539	4.17	1
	B	0.19	0.669	0.12	0.3692	16	0.80199	4.54	0.99
	M	5.948	0.028	0.284	0.6298	16	0.85805	4.54	1
Matchboxes Scotland*	All	11.33	<0.005	0.34	0.72	24	0.82	4.30	1.00

ANOVAs were calculated for both visual conditions and corresponding results of post-hoc power analyses of MGA were computed with G*power 3. The effect size was computed using the actual partial η^2^ of our data for the binocular and monocular condition and for the partial η^2^ that was reported by McIntosh & Lashley [11].Power analyses are based on the a priorily chosen α error probability threshold of 0.05. *data from McIntosh & Lashley [11].

To account for the actual metrical differences between box location changes and box height changes (9 cm versus 9 mm), which might have biased statistical effect size, we quantified additionally the relative change in depth displacement and grip aperture. Not only the absolute but also the relative biases are smaller for the MGA compared to depth displacement. We further inspected the relative differences between the perturbation and baseline trials for depth displacement (relDiffDD) and grip aperture (relDiffMGA) using an analysis of variance. This 2 (relDiffDD vs. relDiffMGA, within-subject)×2 (direction of perturbation, within-subject)×2 (viewing condition, between-subject) ANOVA revealed a significant difference between the relDiffDD and the relDiffMGA (F(1,30) = 13.11, p = 0.001). The results for the perturbation induced bias for the depth displacement and the MGA are shown in figures 3 and 5, respectively.

## Discussion

We conducted the present study to disentangle the contribution of mere perceptual features on the one hand and pre-experimental object familiarity on the other hand to the establishment of object-size associations that influence action implementation. Therefore, we used the same objects as McIntosh & Lashley [11], but in a population that was for sure not familiar with these matchboxes.

In summary, we found a strong and highly significant effect of familiar size for reach distance not only in the monocular but also in the binocular viewing condition. Comparisons of the results from the present study with those from the previous one using the coloured cuboids [12] revealed increased effects, metrically and statistically, in both viewing conditions when we used the matchboxes (see Figure 3). These results of the depth displacement biases argue in favour of the visual saliency as an important key feature to establish a reliable association of the boxes and corresponding sizes. Long-term familiarity is not needed to evoke an effect of illusionary sizes in the reaching phase in the binocular condition. Rather the visual complexity, and thus the distinctness, of the two objects is already sufficient to influence the reaching component of visuomotor control.

However, the effects on depth displacement were not equally pronounced in the two manipulations in the binocular condition. The effect was amplified for the perturbation with the small replica of the originally big *Swan* box, participants overshot more than they undershot for the big replica of the originally small *Bluebell* box. This asymmetric effect could be explained by the use of online corrections during the reaching phase. Whilst the subject still has not touched the target object, the movement can easily be extended. But such an online correction is not possible anymore once the participant already hit the box that was expected to be farther away. In the monocular viewing condition, on the other hand, the undershoot is metrically larger than the overshoot. This reversal in comparison to the binocular conditions might be due to two factors. First, participants without stereovision cannot reliably judge distances in our paradigm and thus are less able to correct their movements online based on updated information during the reach towards the object. Furthermore, in the presence of much larger effects in comparison to the binocular condition it is simply not possible to over-reach in the near perturbation trials as much as it is possible to under-reach for the far perturbation trials. At a certain point the participants simply bump into the presented object.

Altogether, our results support the idea of a modified weak cue fusion model as proposed by Landy and colleagues [7] for the combination of multiple depth and size cues. The stronger the associations between particular objects and their sizes are the more reliable becomes the pictorial size cue being capable to compete with the highly reliable cues obtained during binocular vision. Apparently the visual complexity, i.e., the number and saliency of distinguishable visual features of the matchboxes is sufficient to form a strong object-size association under binocular viewing conditions without any prior experience with these objects in our experiment. This observation and conclusion is further confirmed by the informal personal reports of our participants in the two experiments. When asking the subjects at the end of the experiment, how many different objects they have grasped during the experiment, most of them were able to tell how many boxes were used (median responses: binocular condition: 4 boxes, monocular condition: 3 boxes). More interestingly overall 7 (5 binocular, 2 monocular) participants actively reported that they noticed the change in sizes of the boxes during the perturbation trials. This is in contrast to the previous experiment using the coloured cuboids, where none of the participants consciously detected and reported the manipulation in the last two trials of the experiment. Although we cannot address the obvious question whether the observed effects under binocular conditions depend on the overt detection of differences between the boxes, this anecdotal evidence shows that the distinctiveness of the matchboxes was clearly higher in comparison to uni-coloured boxes.

While the results for the reaching component of the grasp in terms of the depth displacement showed significant effects, we could only find a clear influence of the associated size on the grasp pre-shaping as measured by the MGA under monocular vision. Under preserved stereovision the grasping component of the movement seemed to be less susceptible to the influence of illusionary sizes. Apparently our following assumptions here are primarily based on null-observations. To exclude that we failed to observe any effect of familiar size on the MGA due to low power, we conducted a power analysis. This analysis revealed a very high (99%) overall power. Further, we detected strong and consistent effects for the factor physical size on the MGA in the binocular condition. These results suggest that it is rather unlikely that we failed to detect an actual effect only due to low power.

Comparing the effect sizes for the depth displacement and the MGA reveals that even the effect size for the physical size is larger for the depth displacement (η^2^ = 0.70) than for the MGA (η^2^ = 0.15). One possible explanation might lie in the metrical differences of location distances and box heights. Whilst the two locations for the baseline and perturbation trials were 9 cm apart from each other, the original boxes and their corresponding replica differed only by 9 mm in height. This allows for metrically larger effects that consequently might even result in statistically stronger effects for the reaching distance than for the MGA. However, the experimental question we addressed in this study required that the presentation of the original boxes in the baseline trials and the replica in the perturbation trials elicit the same retinal image to induce the illusionary size. Within these experimental constraints it is not feasible to create graspable boxes that change by the same amount in height as they differ in reaching distance. By reanalysing the effects in relative rather than absolute measures, we nevertheless verified that the MGA was less affected by the pictorial size cue than depth displacement.

The conflicting results of the depth displacement and the MGA indicate functional differences in the influence of pictorial size cues on the reaching and grasping component of the movement. Support for a dissociated influence of pictorial cues on the different components of grasping comes from studies investigating the influence of visual illusions on grasping. Using the Ponzo illusion, Brenner & Smeets [15] showed that illusionary size had no influence on grip scaling, but changed the velocity profile of the movement. Changes of the velocity profile, but not of the MGA, were also reported by Westwood and colleagues [16]. Similarly Jackson & Shaw [17] demonstrated an influence of pictorial cues on grip force, whilst the grip aperture remained unaffected. Support for an only limited influence of size cues on grasping comes also from a set of experiments from Haffenden & Goodale [18], [19]. They nicely showed that an influence on the MGA was only present if the respective size cue was prominent enough as covering the whole surface of the object. Simple symbols, that actually made the objects distinguishable enough to influence perception, did not influence grip scaling. Long-term familiarity instead can influence grasping even on a multisensory level. Parma et al. [20] showed a priming effect of familiar flavours on the grasping of fruits. The scaling of MGA was biased according to the flavour of a solution (e.g. strawberry or apple) that the participants drank prior to grasping the real objects [20]. These assumptions of influences of familiarity on grasping are further supported by a patient study. Patient A.T. suffering from bilateral parietal lesions showed impaired hand pre-shaping when she was asked to grasp featureless cylindrical objects. However, when she was grasping real objects that she was familiar with, such as a lipstick, she improved her grip-scaling according to the objects’ sizes [21].

When investigating size and distance estimations in the framework of the size-distance invariance hypothesis, Haber & Levin [22] reported that familiar size information improved distance estimation when distance information was inadequate. Kaufman et al. [23] found that objective size discrimination is noisier than depth discrimination suggesting that distance is processed prior to angular size. Haptically manipulating felt distance or object size in a reach-to-grasp paradigm, a moderate degree of cross-coupling between reaching and grasping components was found [24]. This coupling was dependent on the strength of the parameter, i.e., whether the object size/distance was increased or decreased and in which direction. Though they reported some measure of independence between reaching and grasping components, they also found clear interaction effects. As for our experiment, we would hypothesise that distance and size estimations were tightly coupled due to the invariance hypothesis and the distinctive objects that we have used. Nevertheless, considering the findings from Kaufman et al. [23], subjects could have relied more on distance information than on object size processing when programming their action, since reaching the object is rather a precondition for successfully grasping it. Since Coats et al. [24] have reported some directional cross-coupling of both distance and size estimation, the more precise distance programming could have overhung the size programming, which might have reduced the familiar size effect for the MGA.

Note that the observed familiar size effect could as well be considered as a conditioned motor response, i.e., as an automatic linkage activation between the sensorimotor subsystems as was suggested e.g. for prism adaptation [25]. The effects on reach (under monocular und binocular viewing) and grasp computations (under monocular viewing) could be equally explained by two models: 1) the pictorial information could be perceived and directly result in a conditioned motor response (linkage activation, [25]), or 2) the pictorial information could be perceived and evaluated resulting in a size-object association, which is then used to compute a motor response. The differentiation between these two possible mechanisms is a very interesting research question in its own rights. However, the primary aim of the current study was to investigate under which presentation and learning conditions an effect of object-size association can be observed at all.

To conclude, the present study dissociates the influences of purely visual features of an object and potential familiarity of participants with the respective object on the reaching and grasping component of visuomotor control. Whilst reaching is influenced reliably by a size-object association that is formed in a short-term acquisition based on the distinctiveness of the stimuli, grasping needs stronger associations that cannot be that easily formed during short-term learning.
